# Risk at the Crossroads: Evaluating the Risk of Sexually Transmitted Infections in High-Risk Groups

**DOI:** 10.7759/cureus.86459

**Published:** 2025-06-20

**Authors:** Vijaylakshmi Jain, Sumit Aggarwal, Kuldeep Nigam, Bhushan Kamble, Rashmi Kundapur, Deepthi Konda, Mishu Mangla, Raja Sundaramurthy, Meely Panda, Lakshmi Jyothi Tadi

**Affiliations:** 1 Department of Community and Family Medicine, All India Institute of Medical Sciences, Bibinagar, Bibinagar, IND; 2 Division of Descriptive Research, Indian Council of Medical Research, New Delhi, IND; 3 Department of Dermatology, All India Institute of Medical Sciences, Bibinagar, Bibinagar, IND; 4 Department of Obstetrics and Gynaecology, All India Institute of Medical Sciences, Bibinagar, Bibinagar, IND; 5 Department of Microbiology, All India Institute of Medical Sciences, Bibinagar, Bibinagar, IND

**Keywords:** high-risk groups, india, reproductive tract infections, risk assessment, sexually transmitted infection

## Abstract

Background: Sexually transmitted infections (STIs) continue to pose a serious public health challenge globally, including in India. With over one million new cases reported annually, inadequate testing and screening remain significant barriers to controlling and ultimately eliminating STI transmission. Men who have sex with men (MSMs), transgender individuals (TGs), female sex workers (FSWs), and injecting drug users (IDUs) are widely recognized as experiencing higher infection rates. In this context, we aimed to evaluate STI-associated risk factors among high-risk group (HRG) communities in Hyderabad.

Methods: A community-based, cross-sectional study was conducted from February 2024 to January 2025 to assess STI risk across high-risk groups, including IDU, MSM, FSW, and TG populations residing in various parts of Hyderabad city. A questionnaire-based STI risk assessment tool, adapted from NACO guidelines, was used in the study. Participants were administered the standardized tool, and pertinent sociodemographic data were collected and analyzed using Jamovi v2.6 software.

Results: The study employed a questionnaire-based tool to identify and address key socio-behavioral risks associated with STI transmission. A significantly high risk of STIs was observed across all typologies, with particularly elevated risk in MSM (87.3%) and TG (69.2%) individuals falling into the medium-to-high risk category. Substantial risk was also noted in FSWs (57.5%) and IDUs (60.7%), primarily under the low-risk category. Factors such as younger age, marital status, and family structure were associated with higher STI risk.

Conclusion: A significantly higher risk of STI was observed among MSM and TG individuals compared to FSWs and IDUs within the overall HRG population. These findings highlight the utility of STI risk screening in targeted intervention sites, NGOs, and healthcare settings. They further emphasize the need for focused interventions and routine STI screening, particularly among high-risk groups.

## Introduction

Sexually transmitted infections (STIs)/reproductive tract infections (RTIs) have become major contributors to stigma, illness, sexual dysfunction, infertility, and negative health outcomes worldwide. Beyond causing acute health problems, they are also linked to serious complications such as congenital abnormalities, infertility in both men and women, and premature births [1]. According to a 2018 WHO report [2], STIs remain a critical global public health concern, leading to approximately 2.5 million deaths annually and around 357 million new cases. Each day, about 1 million individuals aged 15-49 contract an STI, with most infections being asymptomatic [3,4].

In India, STIs affect an estimated 4% of the general population and 6% of high-risk groups (HRGs), translating to approximately 30-35 million STI/RTI episodes annually [5,1]. In 2018, Hyderabad, Telangana, reported the highest rates of gonorrhoea (6.4%) and chlamydia (6.5%), along with a rising trend of syphilis at 17.4% [1,3]. Certain populations, such as men who have sex with men (MSMs), transgender individuals (TGs), female sex workers (FSWs), and injecting drug users (IDUs), are more vulnerable due to high-risk sexual behaviors, including having multiple sexual partners and engaging in unprotected sex, often for financial reasons.

Challenges such as limited access to affordable STI diagnostic services at the primary care level, the asymptomatic nature of many infections, and insufficient specific data have prompted the WHO to develop the STI Spectrum Model. This model serves as a comprehensive conceptual and epidemiological framework that captures the range of STIs across their clinical, diagnostic, and public health dimensions. It helps countries, particularly low- and middle-income nations, estimate STI prevalence and strengthen prevention and treatment efforts [2,6,7].

To promote early detection and treatment, risk assessment tools have been developed to help individuals evaluate their risk of STIs/RTIs. Various online and offline tools, such as SexPro, MySTIRisk, and the San Diego Early Test, use different interfaces to assess STI risk [8]. In India, a 13-item questionnaire developed in line with NACO guidelines serves as a tool to estimate an individual’s risk of contracting an STI/RTI. Positive screenings are followed by clinical validation and contact tracing.

This study aims to assess STI risk in HRGs, namely, IDU, MSM, FSW, and TG, categorized as low and medium to high in community settings, and to highlight the value of risk assessment tools in facilitating early diagnosis and targeted interventions for those at greater risk.

## Materials and methods

Study design and study setting

A community-based cross-sectional study was conducted within the HRG community, focusing on IDUs, MSMs, FSWs, and TGs. The study was conducted in community-based TI sites working with the HRG population in and around the Hyderabad region of Telangana, from February 2024 to January 2025.

Study population

The study included selected HRGs registered at the targeted intervention (TI) sites under NACO/TSACS, including IDU (injecting drug users), MSM (men-having sex with men), FSW (female sex worker), and TGs (transgenders).

Sample size and sampling technique

Sample size was calculated using OpenEpi, based on the proportion of STIs in different HRGs (p), with a 95% confidence level, an absolute error of 5%, and a 20% non-response rate. The final number of participants from each typology was: IDU, 28; MSM, 103; FSW, 120; and TG, 91. Consecutive sampling was used to enroll participants at TI sites until the required sample size for each HRG group was achieved. This tool, derived from NACO guidelines, has not yet been validated for broader application across diverse settings and may be subject to social desirability bias in self-reported responses. It requires field testing and is designed with sufficient flexibility to accommodate regional variations in prevalence rates.

Inclusion and exclusion criteria

The inclusion criteria included the following: (1) age above 18, (2) those fulfilling case definitions of the specific typology, and (3) consent to participate in the study. The exclusion criteria included the following: (1) uncooperative participants and (2) those seriously ill and unable to comprehend the questionnaire.

Risk assessment score

A 13-question risk assessment-based questionnaire, adapted from the NACO, Targeted Interventions Under NACP III: Core High Risk Groups guidelines, 2007 [9] (Table 1), was used in the study to assess STI risk. Along with the risk score, socio-behavioural characteristics such as age, religion, marital status, education, sexual orientation, sexual behaviour method, condom usage, number of sexual partners, comorbidities, and living set-up were recorded.

In-person interviewer-administered questionnaire sessions were conducted. The questionnaire was available in both English and the local language, Telugu. Interviewers were trained and proficient in Telugu, Hindi, English, and Marathi. As most participants were Telugu-speaking, additional language training for administering the questionnaire was provided to the staff.

The risk status (as per Table 1) was determined as follows: low risk, 0-2; medium risk, 3-7; and high risk, 8-13. 

**Table 1:** Risk score based assessment tool for HRG population HRG: high-risk group.

Parameter	Criteria	Score
Condom not used in the last 10 sexual acts	Yes/No	1/0
Condom compromise in the last 10 sexual acts	Yes/No	1/0
Condom breakage in the last 10 sexual acts	Yes/No	1/0
Anal sex	Yes/No	1/0
Lubrication use in the last 10 sexual acts	Yes/No	1/0
First year in sex work	Yes/No	1/0
Below age 25 years	Yes/No	1/0
STI history past 3 months	Yes/No	1/0
Alcohol use (before and during sex)	Yes/No	1/0
Unsafe sex (more money) in the last 10 sexual acts	Yes/No	1/0
Sexual violence	Yes/No	1/0
Migration in the past 3 months	Yes/No	1/0
Group sex in the past 3 months	Yes/No	1/0
Total Risk Assessment Score		13

Ethical clearance

Ethical clearance was obtained from the Institutional Ethics Committee (IEC) of All India Institute of Medical Sciences (AIIMS), Bibinagar (No: AIIMSIBBN/IEC/NOV/2023/342, dated 16/11/23), and administrative approval was secured from NACO, TSACS, and the respective TI sites. Written informed consent was obtained from all participants in their local language after the study procedure was explained to them. Data confidentiality was strictly maintained. Participants identified as high risk were referred to the dermatology OPD at AIIMS Bibinagar or a nearby government-designated DSRC for further evaluation.

Statistical analysis 

All data were entered into MS Excel (Microsoft Corporation, Redmond, Washington) and analyzed using Jamovi statistical software version 2.6 (jamovi Project, Sydney, Australia) [10,11]. Descriptive statistics were calculated, including mean ± standard deviation for continuous variables, and frequencies and proportions for qualitative variables. Independent t-tests and chi-square tests were applied, and bivariate analysis was performed. A p-value of less than 0.05 was considered statistically significant.

## Results

Risk level in HRGs

**Figure 1:**
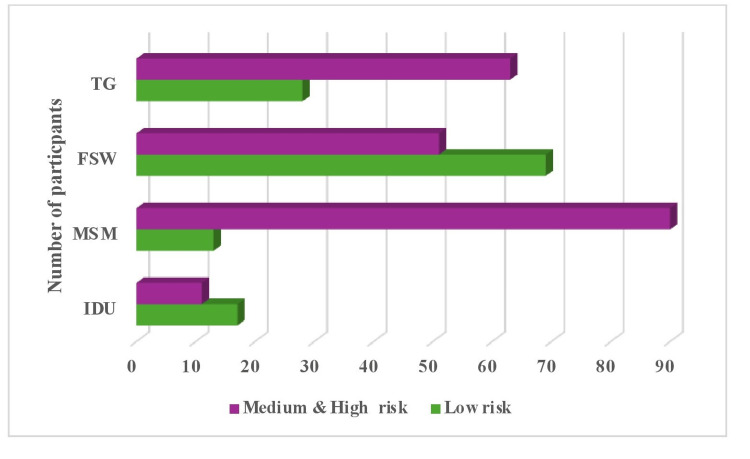
Risk categories based on typologies in HRGs (n=342) MSMs: men who have sex with men, TGs: transgender individuals, FSWs: female sex workers, IDUs: injecting drug users, HRG: high-risk group.

A total of 342 HRG individuals belonging to four typologies, such as IDU (28), MSM (103), FSW (120), and TG (91), aged between 18 and 65 years, participated in this study conducted in Hyderabad, Telangana. The mean age (mean ± SD) of the participants was 31.1 (± 3.4) years. Out of 342 participants, more than half (62.9%) belonged to the medium-to-high-risk category, and 37.1% of participants belonged to the low-risk category. Risk assessment scores classified all four typologies as low risk (0-2), medium risk (3-7), and high risk (8-13). The majority (60.7%) of IDUs were at low risk, while 39.3% were at medium risk. Among 103 MSM, 12.7% were low risk, while 87.3% were medium risk. Out of 120 FSWs, nearly half (57.5%) were at low risk, and 42.5% were at medium to high risk, and the majority of TG (69.2%) were at medium to high risk (Figure 1).

Sociodemographic characteristics in HRGs

Table 2 outlines the basic socio-demographic characteristics of the HRG participants, including their age, marital status, living setup, co-morbidities, education, sexual behavior method, alcohol consumption, number of sexual partners, and mean age of first intercourse. More than half of the population was above the age of 25 years (220; 64.3%), where IDUs represented the younger population aged between 18 and 32 years. TGs and MSMs spanned both younger and older age groups (18-65), while FSWs mostly fell within the 25 to 40 years range, with some outliers. In this study, nearly one-third of the participants (35%) (120 FSWs) were female, 26.7% were TGs, and the remaining 38.3% were male (IDUs and MSMs).

The majority (62.6%) of HRG participants were either unmarried, separated, or widowed. Among subgroups, most (89.3%) of IDUs were unmarried, followed closely by MSMs and TGs, with nearly 90% of each group remaining unmarried. In stark contrast, out of 120 FSWs, most (85%) were married, constituting 79.7% of married individuals within HRGs. Living arrangements significantly influence mental and behavioral well-being. The majority (66.3%) of HRGs resided with family, notably including all IDUs and 90.8% of FSWs. Conversely, around one-third (33.7%) lived alone or with a partner, with TGs representing over 50% of this group (67%), followed by MSMs (41.7%).

Among the 342 participants, most (78.9%) were literate; however, 64.4% of the 227 literate individuals were categorized as higher risk. This suggests that literacy alone may not be protective against high-risk behaviors among MSM and TG individuals in this cohort (Table 2).

Sexual behavior patterns revealed diverse practices. MSM participants predominantly practiced anal sex (40.3%) and oral sex, while TGs (77.4%) engaged exclusively in oral and anal intercourse. No vaginal intercourse was reported among TGs, as all belonged to the Kothi sub-group of transgenders.

MSM and TGs are considered at higher risk due to factors such as early initiation of sexual activity, by the average age of 14 years, followed by IDUs at 16 years, and FSWs by 17 years. TGs, followed by MSMs, had the highest number of sexual partners per week, 6 ± 8.8 and 5 ± 5.1, respectively. Alcohol consumption was reported by 60.4% of HRGs out of 342, of whom 66.4% belonged to the medium-to-high-risk group. This was most prevalent among IDUs (100%), followed by MSMs (61.2%), TGs (60.4%), and FSWs (49.2%) (Table 2).

**Table 2:** Sociodemographic and behavioral characteristics of HRGs in respective typology *Multiple responses possible. MSMs: men who have sex with men, TGs: transgender individuals, FSWs: female sex workers, IDUs: injecting drug users, HRG: high-risk group.

Characteristics	IDU (N=28), n (%)	MSM (N=103), n (%)	FSW (N=120), n (%)	TG (N=91), n (%)	Total (N=342), n (%)
Age in years					
<25	23 (82.1)	43 (41.7)	21 (17.5)	35 (38.5)	122 (35.7)
>25	5 (17.9)	60 (58.3)	99 (82.5)	56 (61.5)	220 (64.3)
Marital status					
Married	3 (10.7)	18 (17.5)	102 (85)	5 (5.5)	128 (37.4)
Unmarried/widow/separated	25 (89.3)	85 (82.5)	18 (15)	86 (94.5)	214 (62.6)
Living set-up					
Family	28 (100)	60 (58.3)	109 (90.8)	30 (33)	227 (66.3)
Alone/partner	0 (0)	43 (41.7)	11 (9.2)	61 (67)	115 (33.7)
Co-morbidities					
Yes	2 (7.1)	24 (23.3)	42 (35)	11 (12.1)	79 (23.1)
No	26 (92.9)	79 (76.7)	78 (65)	80 (87.9)	263 (76.9)
Education					
Literate	27 (96.4)	98 (95.1)	74 (61.7)	71 (78)	270 (78.9)
Illiterate	1 (3.6)	5 (4.9)	46 (38.3)	20 (22)	72 (21.1)
Alcohol consumption					
Yes	28 (100)	63 (61.2)	59 (49.2)	55 (60.4)	205 (60)
No	0 (0)	40 (38.8)	61 (50.8)	36 (39.6)	137 (40)
Sexual behavior method*					
Vaginal	28 (15.9)	28 (10.8)	120 (63.1)	0 (0)	176 (51.4)
Anal	2 (1.3)	86 (40.3)	19 (9.7)	75 (48.7)	182 (53.2)
Oral	2 (3.2)	88 (77.4)	9 (19.4)	91 (100)	190 (55.5)
Mean age (mean ± SD) of having first intercourse in years	16 ± 4.9	14 ± 4.4	17 ± 4	14 ± 3.4	NA
Mean number of sexual partners/week (mean ± SD)	2 ± 1.9	5 ± 5.1	3 ± 1.1	6 ± 8.8	NA

Sexual practices and risk status in HRGs

**Figure 2:**
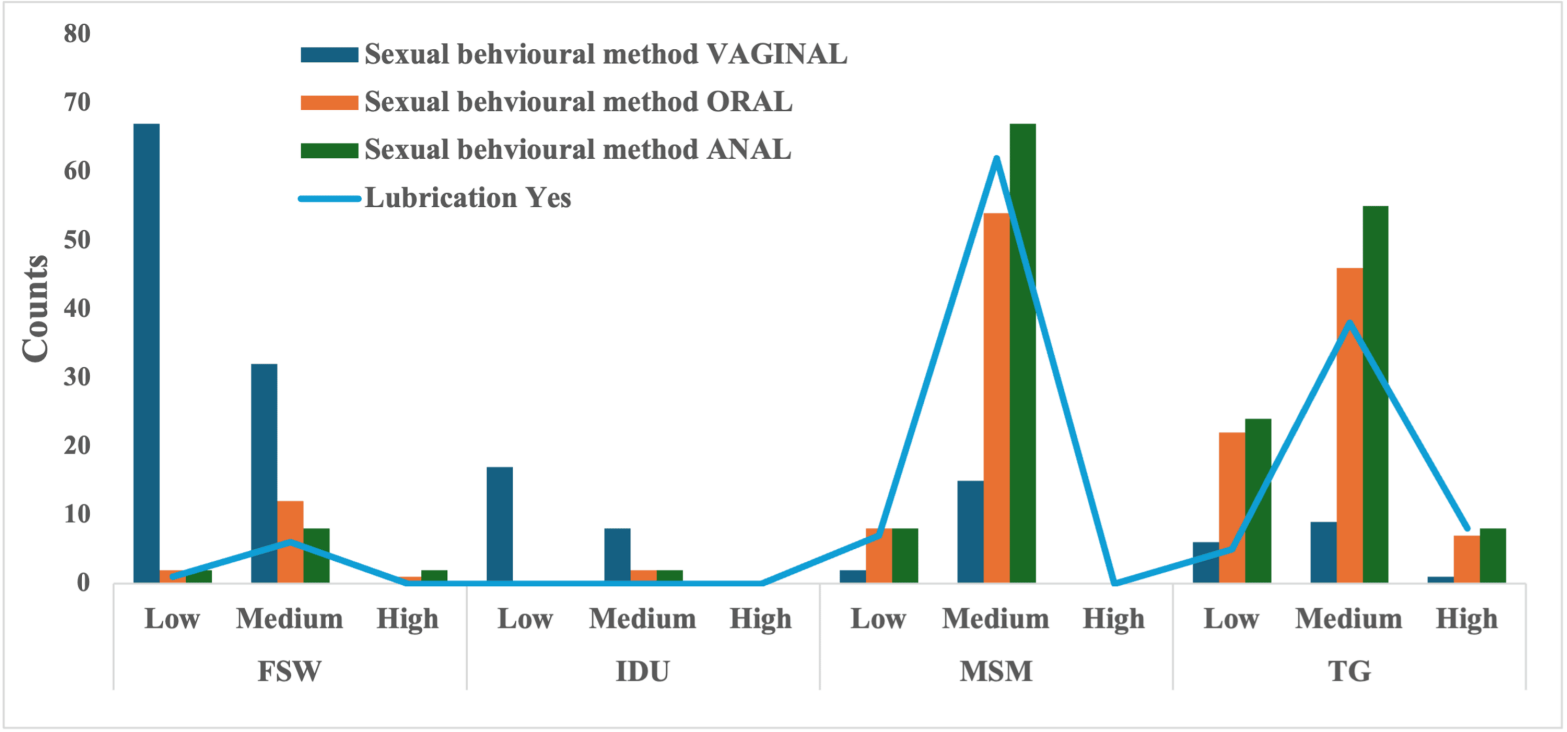
Sexual behavioral methods and risk status among HRGs MSMs: men who have sex with men, TGs: transgender individuals, FSWs: female sex workers, IDUs: injecting drug users, HRG: high-risk group.

HRGs reported varied sexual practices, with 90.3% engaging in oral sex, 79.2% in anal sex, and 41.4% in vaginal sex. Anal sex (p < 0.001; OR: 5.3, CI: 3.2-8.9) and oral sex (p < 0.001; OR: 13.2, CI: 3.8-45.2) were significantly associated with medium to high STI risk. Among TGs, 100% reported engaging in anal sex, yet only 48.3% reported consistent condom use, with high partner numbers further compounding the STI risk (p < 0.001) (Figure 2). 

Condom use, compromise, and risk status in HRGs

**Figure 3:**
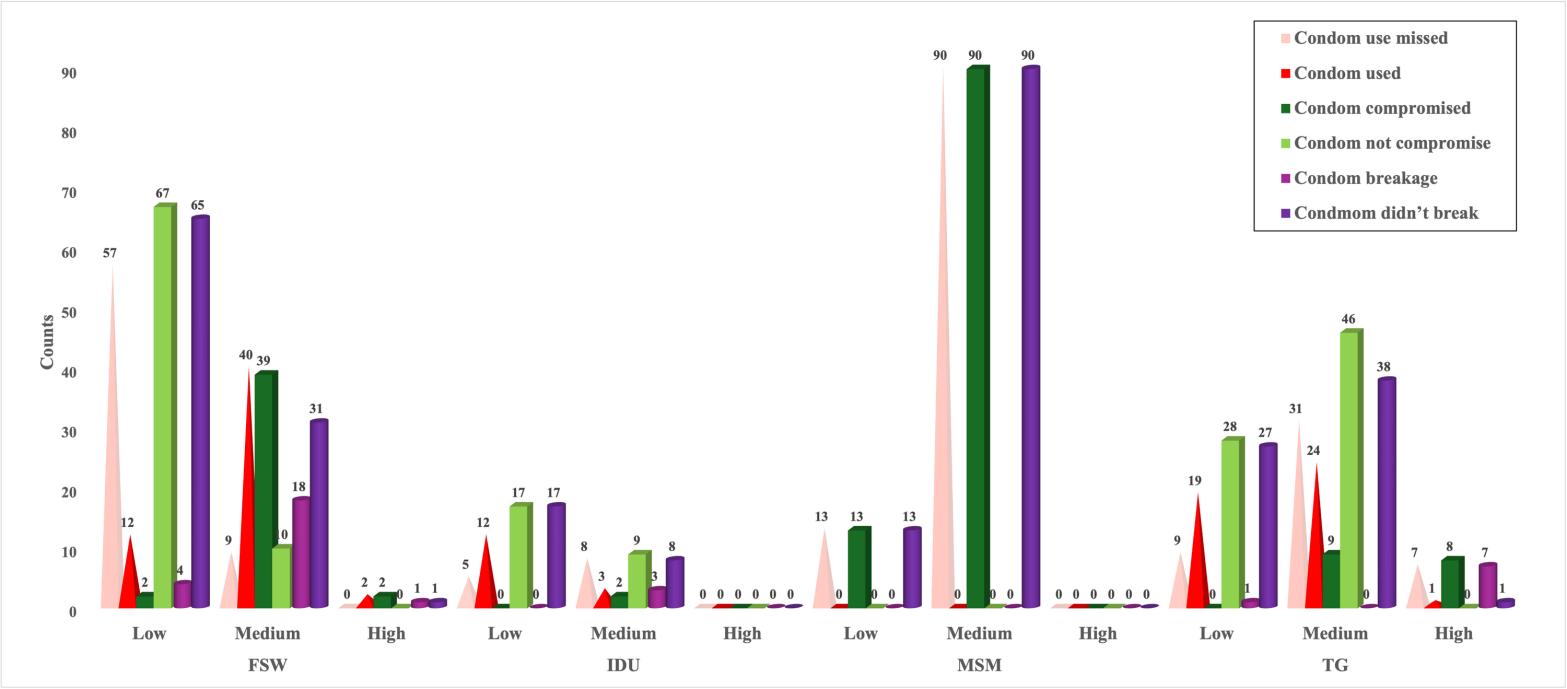
Condom use, breakage, and compromise affecting the risk status of HRGs HRG: high-risk group.

MSMs reported high rates of anal sex (83.4%) and condom use (87.3%); however, all participants reported experiencing condom failure, placing them at the highest STI risk. This suggests that condom use alone may be insufficient, possibly due to factors such as high partner turnover and frequent condom compromise. A similar pattern was observed among TGs, placing them as the second-highest risk group. A chi-square test confirmed a significant association between condom compromise and STI risk (p < 0.05) (Figure 3).

Risk profiling of HRGs

On bivariate analysis, we found that younger age (<25 years), marital status, living arrangement, and sexual behavioral practices were significantly associated with STI risk status. Among participants under 25 years, only 35.7% were classified as low risk, compared to 64.3% of those aged 25 and above. Notably, 73% of the 122 younger individuals were at medium to high STI risk. These findings suggest that younger participants were significantly more likely to be at elevated risk than older adults (p < 0.004; OR = 2.01, 95% CI: 1.2-3.2). Among HRGs, 62.6% (214) were unmarried, widowed, or separated, of whom 71% were at higher risk and 29% were at lower risk. Thus, marital status significantly influenced STI risk (p < 0.001; OR = 2.5, 95% CI: 1.6-3.9).

Living alone or with a partner was associated with a higher risk compared to living with family, based on data from 115 (33.7%) participants. Of these, the majority (76.5%), mainly MSM and TG individuals, were at higher risk (p < 0.001; OR = 2.5, 95% CI: 1.5-4.5). HRGs reported varied sexual practices, with 90.3% engaging in oral sex, 79.2% in anal sex, and 41.4% in vaginal sex. Anal (p < 0.001; OR = 5.3, 95% CI: 3.2-8.9) and oral sex (p < 0.001; OR = 13.2, 95% CI: 3.8-45.2) were significantly associated with higher STI risk. Anal sex poses heightened risk due to condom failure, lubricant use, and increased fluid exposure. Among transgender individuals, 100% reported anal sex, yet only 44% used condoms consistently, and high partner numbers further compounded the STI risk (Table 3).

**Table 3:** Association of STI risk with sociodemographic and behavioral factors among HRG using bivariate analysis Ref.: reference, OR: odds ratio, CI: confidence interval, STI: sexually transmitted infection, HRG: high-risk group.

Characteristics	Low risk (N=127), n (%)	Medium and high risk (N=215), n (%)	Total (N=342), n (%)	p-value	OR (95% CI)
Age in years					
>25	94 (42.7)	126 (57.3)	220 (64.3)		Ref.
<25	33 (27)	89 (73)	122 (35.7)	<0.004	2.01(1.2-3.2)
Marital status					
Married	65 (50.8)	63 (49.2)	128 (37.4)		Ref
Unmarried/widow/separated	62 (29)	152 (71)	214 (62.6)	<0.001	2.5 (1.6-3.9)
Living set-up					
Family	100 (44.1)	127 (55.9)	227 (66.3)		Ref
Alone/partners	27 (23.5)	88 (76.5)	115 (33.7)	<0.001	2.5 (1.5-4.5)
Comorbidities					
Yes	35 (44.3)	44 (55.7)	79 (23.1)		Ref
No	92 (35)	171 (65)	263 (76.9)	0.133	1.49 (0.88-2.46)
Education					
Literate	96 (35.6)	174 (64.4)	270 (78.9)		Ref
Illiterate	31 (43.1)	41 (56.9)	72 (21.1)	0.242	0.730 (0.4-1.2)
Alcohol consumption					
Yes	69 (33.7)	136 (66.3)	205 (60)		Ref
No	58 (42.3)	79 (57.7)	137 (40)	0.104	0.69 (0.4-1.08)
Alcohol use (before and during sex)					
Yes	35 (27.6)	76 (35.3)	111 (32.5)		Ref
No	92 (72.4	139 (64.7)	231 (67.5)	0.137	0.69 (0.4-1.1)
Sexual behavior method		,			
Vaginal	92 (58.6)	65 (41.4)	157 (45.9)		Ref
Anal	32 (9.7)	122 (79.2)	154 (45)	<0.001	5.3 (3.2-8.9)
Oral	3 (9.7)	28 (90.3)	31 (9.1)	<0.001	13.2 (3.8-45.2)

## Discussion

This study utilized a structured questionnaire to stratify STI/RTI risk levels among key populations, including IDUs, MSM, FSWs, and TGs, who were recruited from TSACS-registered targeted intervention (TI) sites in Hyderabad (N = 342). A substantial proportion (62.6%) of participants were categorized as medium to high risk, underscoring the ongoing vulnerability of these groups to STI acquisition and transmission. This deviation may reflect increased outreach efforts, condom negotiation skills, and targeted education initiatives among FSWs in the Hyderabad region. Conversely, the persistently elevated risk in MSM and TGs suggests persistent structural and behavioral determinants such as stigma, clandestine sexual practices, limited access to typology-specific friendly services, and lower rates of PrEP or routine screening uptake. Comparable studies by Feulner et al. and Latt et al. demonstrated the efficacy of questionnaire-based screening in similar contexts [8,12].

Latt et al. reported elevated STI positivity among MSM and comparably lower rates among FSWs, consistent with our findings, where MSM and TGs exhibited higher composite risk scores, while FSWs showed the lowest STI risk. This pattern was further corroborated by Garofalo et al., affirming its reproducibility across diverse cohorts [8,13].

Previous studies have identified older adults aged 25-40 years and unmarried individuals as predominantly falling within medium to high STI risk categories. For instance, Nayyar et al. and Bala et al. observed elevated STI risk among this demographic group [14,15]. While our findings on lower STI risk among older IDUs and FSWs diverge from some earlier studies, this could reflect differential access to community-based interventions or shifts in risk behavior with age and experience. These observations suggest an opportunity to reallocate resources dynamically, scaling interventions among younger, newly initiated individuals, especially those reporting early sexual debut. Moreover, Paul et al. highlighted that marital status can heighten vulnerability to STIs, citing factors such as restricted sexual autonomy and limited capacity for negotiation within intimate relationships as key contributors [16].

Early sexual debut is a primary determinant of STI risk. We observed that early sexual initiation resulted in medium to high risk of STI, especially in MSM and TGs, followed by IDUs and FSWs. Khezri et al. predicted early sex initiation as a similar finding among FSWs in Iran, consistent with studies on MSM and TGs in the Peruvian population by Castillo et al. [17,18].

Having multiple or more than two sexual partners is strongly associated with increased STI risk, as reported by Wilson Chialepeh and Sathiyasusuman. In our study, medium- to high-risk profiles were most prevalent among MSM and TGs, followed by IDUs and FSWs. These findings are consistent with those of Castillo et al., who observed similar risk associations in MSM, TGs, and FSWs, further corroborating the link between multiple sexual partnerships and heightened STI incidence [18-20].

Education is essential to control the transmission of STIs. According to Alhussaini et al., despite the 84% literacy rate of the research population, the risk was quite high, particularly among MSM and TGs in sociocultural contexts. Low literacy regarding STIs has been noted in numerous studies and is linked to a critical gap in STI prevention [21,22].

The impact of lubricant use on epithelial integrity is linked to increased HIV transmission and pathogen entry, which has been well documented, particularly among MSM and FSWs [23]. Our findings echoed these observations, notably among MSM and TGs, with a smaller representation among FSWs.

Anal and oral sex were significantly associated with medium to higher STI risk, as observed among MSM and TGs in our study. A comparative cross-sectional study conducted in a tertiary care center in India reported similar findings in MSM by Pravitha et al. Another study by Madhivanan et al. on FSWs and MSM also showed similar findings [24,25].

Alcohol use was highest among IDUs, followed by MSM, TGs, and FSWs. However, no significant risk association was observed in our study. Similar to our findings, Wilson et al. found no association between alcohol consumption and STI risk. This may suggest that alcohol acts more as a syndemic factor that exacerbates vulnerability through indirect pathways such as impaired decision-making or reduced condom negotiation capacity, rather than as a direct predictor of STI acquisition. In contrast, Llamosas-Falcon et al. reported a strong association between alcohol consumption and STI risk across high-risk subgroups [26,27].

Condoms have been widely validated as an effective method for reducing the transmission of STIs. Our study revealed a significant association between compromised condom use and breakage and elevated STI risks in HRGs. Comparable findings were reported by Dirisu et al. in Nigeria among key populations such as MSM, TGs, IDUs, and FSWs. Similar conclusions were drawn by Mustanski et al. and further supported by Ankomah et al. in Ghana [20,28-29].

This study must also be interpreted in light of its novelty and limitations. It is the first to include all major HRG typologies, enhancing the external validity of the risk assessment tool. It offers a cost-effective, scalable approach for rapid STI screening in line with current clinical guidelines. The self-administered questionnaire promotes patient engagement, encourages reflection on behaviors, and reduces social stigma. However, as the study relies on self-reported information, we acknowledge potential biases such as recall bias, social desirability bias, Berksonian bias, and limited sensitivity and specificity due to misclassification and underreporting. While this research was conducted on a local scale, a broader clinical rollout would help validate and reinforce the results. In-person testing remains the gold standard. Unadjusted analyses are used to compare the positive rates between the medium to high-risk categories and the PrEP/non-PrEP groups. To verify the results, multivariate analysis that accounts for important confounders is necessary in subsequent research due to the possibility of confounding by variations in demographic and behavioral traits. Even without adjusting for any confounders, the noticeably higher positive rates that are consistently observed in the high-risk categories across infections are noteworthy.

In conclusion, beyond validating the utility of the self-administered tool, our study provides evidence-based direction for stratified STI prevention in HRGs. The findings advocate for a shift from generalized to precision public health strategies, anchored in behavioral risk profiles and supported by community-responsive frameworks. As a next step, implementation science methodologies should be applied to evaluate the real-world effectiveness of these targeted interventions in improving STI outcomes across diverse subgroups. Our findings support the adoption of non-invasive screening methods, particularly for underserved and low-income populations, and provide a foundation for initiating STI pre-exposure prophylaxis in high-risk communities.

## Conclusions

This study demonstrated a markedly elevated risk of STIs among all HRGs, with MSM and TGs exhibiting significantly greater vulnerability compared to FSWs and IDUs, who also face considerable risk. The key determinants of STI risk include younger age, living arrangements, marital status, engagement in anal and oral sexual behaviors, early sexual debut, engagement in unsafe sexual practices, and multiple sexual partners. Furthermore, the study highlights the paradox of persistently high STI risk despite relatively high literacy levels, pointing to the need for broader awareness and generalized health education for effective STI prevention. The risk assessment tool was intended to identify individuals with elevated risk for targeted interventions; however, baseline testing and counseling should be offered to all individuals, irrespective of assessed risk status. To address the challenges faced, support at the policy level is essential to guarantee workforce training, sustained funding, and uniform standards for the provision of STI services. India, being a country with limited resources where vertical programs frequently function in silos, would benefit from integrating STI diagnostic and treatment facilities with current HIV, maternity, and reproductive health programs, along with all DSRCs at the district level. This integration can improve case discovery, lessen stigma, and encourage comprehensive care. Addressing these multifactorial drivers, along with evolving behavioral trends such as dating app usage, PrEP knowledge, and generalized awareness of STIs through ongoing health programs, is essential for curbing STI transmission and advancing health equity among HRGs.
